# Regulation of migration and invasion by Toll-like receptor-9 signaling network in prostate cancer

**DOI:** 10.18632/oncotarget.4197

**Published:** 2015-06-05

**Authors:** Yun Luo, Qi-Wei Jiang, Jie-Ying Wu, Jian-Ge Qiu, Wen-Ji Zhang, Xiao-Long Mei, Zhi Shi, Jin-Ming Di

**Affiliations:** ^1^ Department of Urology, The 3rd Affiliated Hospital of Sun Yat-sen University, Guangzhou, Guangdong, China; ^2^ Department of Cell Biology & Institute of Biomedicine, National Engineering Research Center of Genetic Medicine, Guangdong Provincial Key Laboratory of Bioengineering Medicine, College of Life Science and Technology, Jinan University, Guangzhou, Guangdong, China

**Keywords:** TLR9, migration, invasion, prostate cancer

## Abstract

Toll-like receptors (TLRs) play an important role in tumorigenesis and progress of prostate cancer. However, the function and mechanism of Toll-like receptor-9 (TLR9) in prostate cancer is not totally understood. Here, we found that high expression of TLR9 was associated with a higher probability of lymph node metastasis and poor prognosis. Further *in vitro* functional study verified that silence of TLR9 inhibited migration and invasion of PC-3 cells, indicating expression of TLR9 involving in the migration and invasion of cancer cells. The data of microarray exhibited silence of TLR9 induced 205 genes with larger than 2-fold changes in expression levels, including 164 genes down-regulated and 41 genes up-regulated. Functional Gene Ontology (GO) processes annotation demonstrated that the top three scores of molecular and cellular functions were regulation of programmed cell death, regulation of locomotion and response to calcium ion. TLR9 signaling network analysis of the migration and invasion related genes identified several genes, like matrix metallopeptidase 2 (MMP2), matrix metallopeptidase 9 (MMP9), chemokine receptor 4 (CXCR4) and interleukin 8 (IL8), formed the core interaction network based on their known biological relationships. A few genes, such as odontogenic ameloblast-associated protein (ODAM), claudin 2 (CLDN2), gap junction protein beta 1 (GJB1) and Rho-associated coiled-coil containing protein kinase 1 pseudogene 1 (ROCK1P1), so far have not been found to interact with the other genes. This study provided the foundation to discover the new molecular mechanism in signaling networks of invasion and metastasis in prostate cancer.

## INTRODUCTION

Prostate cancer is one of the most common solid organ malignancy affecting men in world. Several studies, including meta-analyses, suggest inflammation play an important role on the pathogenesis of prostate cancer [1–3]. Toll-like receptors (TLRs) are evolutionarily conserved transmembrane proteins and critical mediators of the innate immune system, which recognize conserved molecular motifs specific for microbial components to induce various cytokines in regulation of adaptive immune responses [4, 5]. TLR family include kinds of different TLRs, and each of them exhibits an unique expression pattern and recognizes a specific pathogen-derived ligand [6, 7]. For example, TLR4 is expressed on the cell surface and recognize bacterial lipopolysaccharide, while TLR9 resides in intracellular vesicles and is the receptor for bacterial and vertebrate DNA, which contains unmethylated CpG dinucleotide motifs (CpG motifs) [8, 9]. More specifically, TLR7, 8 and 13 are RNA receptors [10]. As reported in the previous study, in immune cells, ligand binding to TLR9 associates with the adaptor, myeloid differentiation primary response 88 (MyD88), and recruits interleukin-1 receptor-associated kinase 1 (IRAK) and TNF receptor-associated factor 6 (TRAF6) to activate the IFN regulatory factor 7 (IRF7), resulting in expression of IFN-alpha, or to activate the IκB kinase complex (IKK) to induce phosphorylation of IκB, resulting in nuclear translocation of NF-κB, which induces expression of inflammatory cytokines [11]. In addition to immune cells, TLR9 is also expressed in various human cancer cells, including prostate cancer. Väisänen MR et al demonstrated that TLR9 is highly overexpressed in prostate cancer in comparison with benign hyperplasia [12]. A high level of TLR9 expression in prostate cancer cells was shown to be significantly associated with a higher Gleason score, a greater probability of biochemical recurrence and poor progression-free survival in patients who were treated by radical prostatectomy [13]. Moreover, the high TLR9 expression was an independent marker of poor prognosis in prostate cancer [14]. Previous studies also showed that treatment of TLR9-expressing prostate cancer cells with synthetic TLR9-ligands, which mimic the structure of bacterial DNA, stimulates the invasion by increasing the matrix metalloproteinase activity *in vitro* [15, 16]. These findings suggest that TLR9 could play a role in the tumorigenesis and progress of prostate cancer. However, the mechanism of cellular DNA receptor TLR9 promoting invasion and metastasis in prostate cancer is still unclear.

In this study, we investigated the expression and clinical significance of TLR9 in prostate cancer tissue and explored the role of TLR9 signaling network in the migration and invasion of prostate cancer. Our study highlighted the mechanism of TLR9 in regulation of migration and invasion of prostate cancer and identified the new targets for anticancer therapeutic intervention.

## RESULTS

### High expression of TLR9 was correlated with a higher probability of lymph node metastasis and poor prognosis of patients with prostate cancer

**Figure 1:**
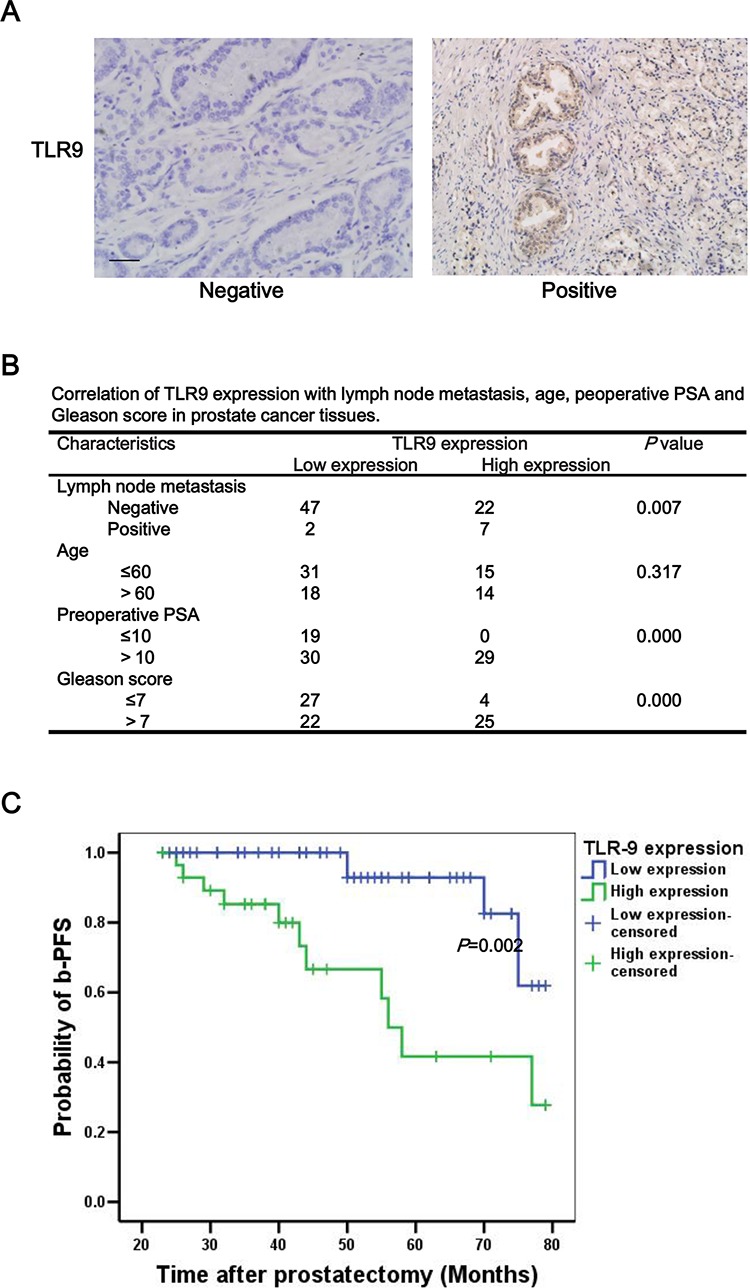
High expression of TLR9 was correlated with a higher probability of lymph node metastasis and poor prognosis of patients with prostate cancer The levels of TLR9 in human prostate cancer tissues were examined by immunohistochemistry staining with anti-TLR9 antibody. The representative TLR9 negative and positive samples **A.** correlation of TLR9 expression with lymph node metastasis, age, preoperative PSA and Gleason scores **B.** and progression-free survival rate of patients with prostate cancer **C.** were shown. Scale bar represents 50 μm.

To investigate the expression of TLR9 in human prostate cancer tissues, a total of 78 prostate cancer specimens were collected and their TLR9 expressions were detected with immunohistochemical staining. The representative results of negative and positive TLR9 expressions were shown in Figure 1A, and the clinical characteristics of patient were summarized in Table 1. The mean age, preoperative (prostate specific antigen) PSA values and Gleason score for patients with low TLR9 expression and high TLR9 expression were 58.55 VS 58.79 years, 12.04 VS 20.18 ng/ml, and 6.20 VS 8.03, respectively. Moreover, 7 cases (24.14%) were positive lymph node metastasis in the 29 cases with high TLR9 expression. Only 2 cases (4.08%) were positive lymph node metastasis in the 49 cases with low TLR9 expression. High level of TLR9 expression was shown to be significantly associated with higher probability of lymph node metastasis, preoperative PSA and Gleason score (Figure 1B). To further determine whether TLR9 expression is associated with prognosis of patients with prostate cancer, progression-free survival rates were compared in patients with high and low TLR9 expression. As shown in Figure 1C, the prostate cancer-specific progression-free survival (b-PFS) rate of patients with low TLR9 expression (*N* = 49) was significantly larger than that of patients with high TLR9 expression (*N* = 29), suggesting the high expression of TLR9 in prostate cancer indicates poor prognosis. Next, Cox multivariate progression- free survival analysis was carried out to examine whether TLR9 expression was an independent factor for predicting prognosis in prostate cancer. We found that positive lymph node metastasis (HR:10.54, 95% CI:2.94–37.80, *P* < 0.001) and preoperative PSA (HR:1.27, 95% CI:1.11–1.46, *P* = 0.001) were independent factors of poor prognosis in prostate cancer, while high TLR9 expression were not an independent factor for predicting prognosis.

**Table 1:** Summary of clinical characteristics and TLR9 expressions of 78 patients with prostate cancer

Characteristics	TLR9 expression		Total *N* = 78
	Low *N* = 49	High *N* = 29	
Age			
Minimum	48	48	48
Maximum	69	69	69
Mean	58.55	58.79	58.64
PSA			
Minimum	4.86	13.22	4.86
Maximum	26.42	29.87	29.87
Mean	12.04	20.18	15.06
Follow-up (M)			
Minimum	23	23	23
Maximum	79	79	79
Mean	51.27	44.31	48.68
Gleason score			
Minimum	3	5	3
Maximum	10	10	10
Mean	6.20	8.03	6.88

### Silence of TLR9 inhibited migration and invasion of prostate cancer

**Figure 2:**
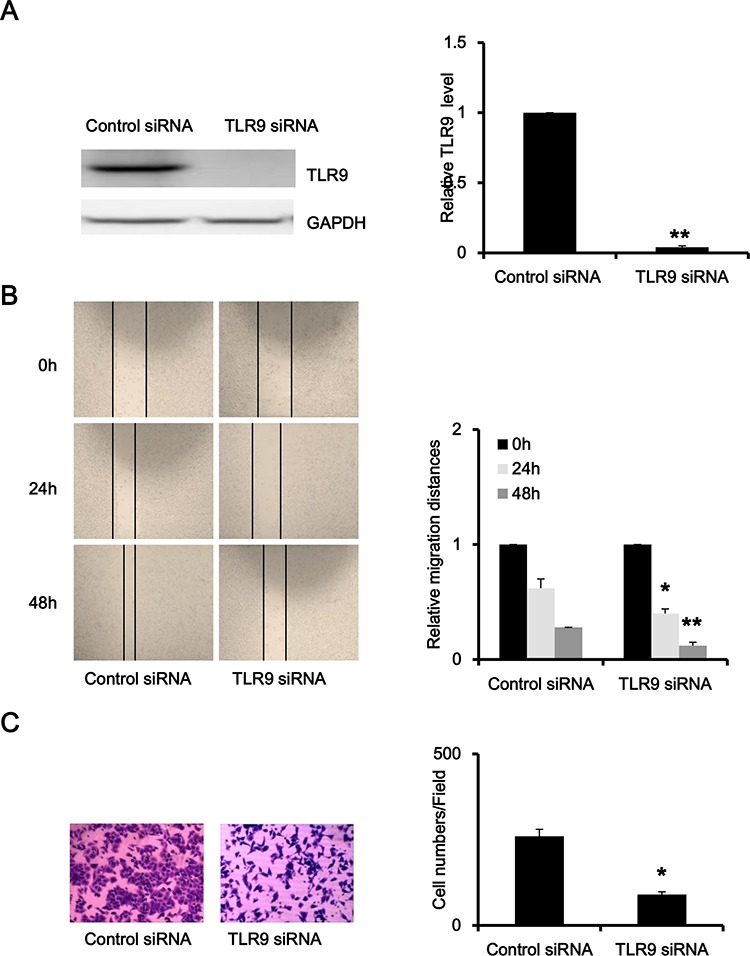
Silence of TLR9 inhibited migration and invasion of PC-3 cells PC-3 cells were transfected with control or TLR9 siRNA. After 48 h, proteins were detected by Western blot with the indicated antibodies. **A.** The scratch-wounds were photographed at the indicated time points (0, 24 and 48 h) after cell scratching and the wound sizes were measured **B.** Cells were seeded into the upper chamber of transwell with matrigel. Cell invasion were induced by FBS-containing media in the lower chamber. Invaded cells in the lower surface of the filters were stained and microphotographed after serum induction 24 h **C.** The representative figures and quantified data from three individual experiments were shown. * and ** represent *P* < 0.05 and *P* < 0.01, respectively.

To explore the function of TLR9 in prostate cancer, we silenced TLR9 expression in PC-3 cells with transfection of TLR9 siRNA. As shown in Figure 2A, TLR9 siRNA significantly silenced TLR9 expression in PC-3 cells in comparison with negative control siRNA, and the efficiency of knockdown was about 90%. We firstly examined the effects of TLR9 silence on cell proliferation, and the results showed that silence of TLR9 did not alter the proliferation of PC-3 cells (data not shown). Next, we evaluated the effects of TLR9 silence on cell migration and invasion. As shown in Figure 2B and 2C, TLR9-silenced PC-3 cells exhibited significant lower potential of migration and invasion in comparison with control cells (Figure 2B). These results indicated that TLR9 might be involved in the migration and invasion of prostate cancer.

### Altered gene expressions of TLR9 signaling network in prostate cancer

**Figure 3:**
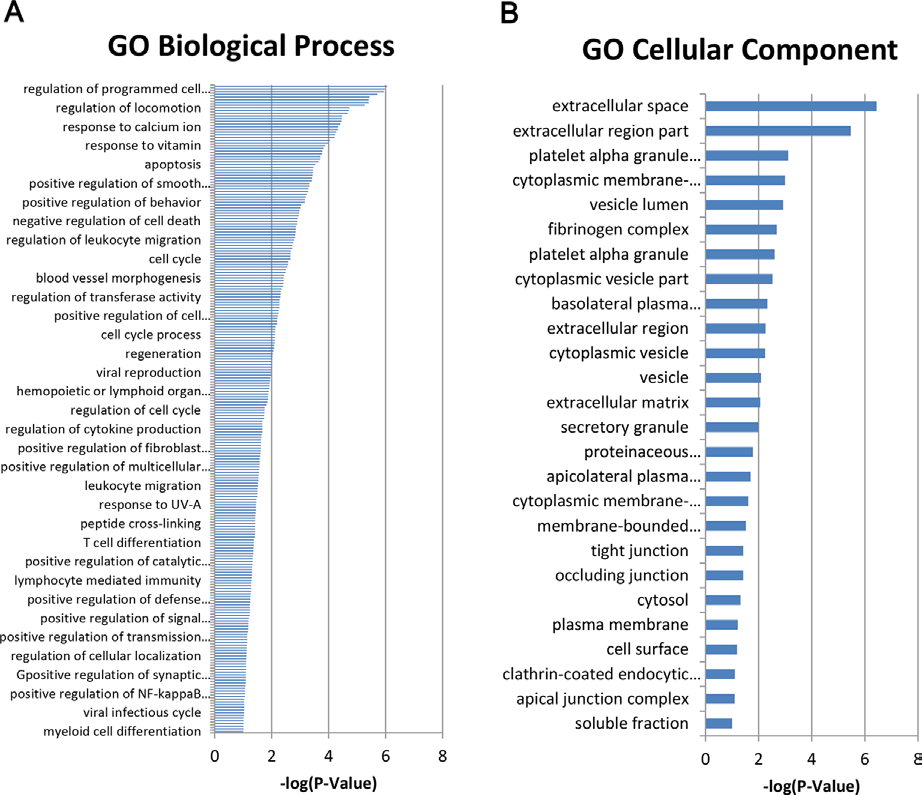
Altered gene expressions of TLR9 signaling network in prostate cancer The GO biological process **A.** and cellular component **B.** of Affymetrix HTA2.0 array. data were anaylsed at http://david.abcc.ncifcrf.gov/.

To better define TLR9 signaling network in both physiological and pathophysiological contexts, we conducted a whole-genome microarray analysis of mRNA levels and examined the fold changes of genes from TLR9-silenced compared with control PC-3 cells. Analysis of the microarray data revealed 205 genes with larger than 2-fold changes in expression levels. The direction of gene alteration was split with 164 genes down-regulated and 41 genes up-regulated (Table 2). Functional Gene Ontology (GO) processes annotation exhibited that the top three scores of molecular and cellular functions were regulation of programmed cell death, regulation of locomotion and response to calcium ion (Figure 3A). In addition, the top three scores of cellular component were extracellular space, extracellular region and platelet alpha granule lumen (Figure 3B).

**Table 2:** Summary of microarray fold-change results between TLR9-silenced and control PC-3 cells

Gene symbol	Fold change	Gene symbol	Fold change	Gene symbol	Fold change	Gene symbol	Fold change	Gene symbol	Fold change
TLR9	0.08	PHLDA1	0.37	GPR126	0.43	HIST1H3F	0.47	GPR110	2.01
CTH	0.10	SIPA1L2	0.37	ANXA1	0.43	KIAA0754	0.47	AGR2	2.01
MMP13	0.12	DDIT3	0.38	COMMD3	0.44	PCK2	0.47	RNY5	2.05
ANKRD1	0.13	TIMP3	0.38	IL18	0.44	SESN2	0.47	GATSL1	2.06
IL8	0.14	CHMP4C	0.38	ERN1	0.44	GABPB1	0.47	SEMA5A	2.09
NOX1	0.16	SOWAHC	0.38	LRCH1	0.44	CAPRIN2	0.47	AKR1C3	2.10
MMP9	0.16	SAT1	0.38	SOX4	0.44	TATDN3	0.47	PLA2G2A	2.11
PTGS2	0.18	STAMBPL1	0.38	PDP1	0.44	PVR	0.47	LRRC61	2.16
PHLDB2	0.18	ADAMTS6	0.39	FAM100B	0.44	PLEKHG1	0.47	SNORD123	2.16
ATF3	0.21	CD55	0.39	RAB8B	0.45	CLIP1	0.47	MX1	2.20
ERRFI1	0.22	LCA5	0.39	PMAIP1	0.45	SLC7A5	0.47	SECTM1	2.22
HIST1H1T	0.22	VEGFA	0.39	EMP1	0.45	SGPP1	0.48	C12orf36	2.23
RGS16	0.23	SPRED1	0.39	TES	0.45	MTHFD2	0.48	SEMA3F	2.23
LIF	0.23	GOT1	0.39	CHIC2	0.45	RND3	0.48	ERP27	2.24
ITGA2	0.25	SIK1	0.39	KCTD20	0.45	ZNF140	0.48	CFH	2.24
COX2	0.26	ROCK1P1	0.39	SIRT1	0.45	CPEB4	0.48	ANK1	2.25
LURAP1L	0.27	TAF1A	0.39	C6orf141	0.45	RHOB	0.48	FGA	2.28
TGFB1	0.27	KLF6	0.39	IER3	0.45	RIPK2	0.48	MAP4K1	2.30
CREB5	0.28	FAM63B	0.39	MT2A	0.45	GADD45A	0.48	PRR15L	2.36
MMP2	0.28	XRCC4	0.39	IZUMO1	0.45	CXCL3	0.48	VGLL1	2.36
RPS6KA5	0.28	CCDC50	0.40	ETV5	0.45	MXD1	0.48	CDH10	2.38
PPP1R15A	0.29	USP54	0.40	AJUBA	0.45	IFRD1	0.48	APOL1	2.43
LDLR	0.30	GTPBP2	0.40	FLJ46906	0.45	KPNA4	0.48	AGMO	2.44
USP53	0.30	PYGB	0.40	GK	0.45	CHAC1	0.48	TMEM27	2.48
RIMKLB	0.31	CCND1	0.40	MET	0.46	XBP1	0.48	PTPN13	2.50
MAP1LC3B	0.31	PARD6B	0.40	BEST1	0.46	C12orf39	0.48	SH3BGRL	2.51
DUSP6	0.32	TNFRSF12A	0.40	CCDC88A	0.46	NAMPT	0.49	REG1A	2.57
HKDC1	0.32	MICAL2	0.40	NEDD9	0.46	C12orf35	0.49	EDAR	2.58
FN1	0.33	PRSS1	0.41	CEBPG	0.46	AMIGO2	0.49	LPCAT1	2.58
NCOA7	0.33	TXNIP	0.41	S100P	0.46	LOC541471	0.49	THBS1	2.65
CDR2	0.33	DDIT4	0.41	PIGA	0.46	TNFAIP8	0.49	GCNT4	2.66
HOXB9	0.33	SKIL	0.42	SERPINB8	0.46	RIOK3	0.49	SPINK1	2.73
TSC22D2	0.34	EPHA2	0.42	ICAM1	0.46	NEDD4	0.49	IGFBP1	2.77
ARRDC4	0.35	CXCL1	0.42	BICD1	0.46	TCP11L2	0.49	GJB1	2.80
NFIL3	0.35	JMJD1C	0.42	C17orf48	0.47	LONRF1	0.49	SULT1E1	2.92
ARRDC3	0.35	LRRC31	0.42	DUSP4	0.47	FOXO3B	0.49	SNCAIP	3.23
MBNL2	0.36	TMEM133	0.42	CLDN1	0.47	ACOT9	0.50	MUC1	3.52
PDGFRA	0.36	GDF15	0.43	CSGALNACT2	0.47	SARS	0.50	RGS5	3.64
TUBE1	0.36	CCDC68	0.43	SKP2	0.47	IER3	0.50	CLDN2	4.31
ARHGEF2	0.36	REG4	0.43	DUSP1	0.47	CLIC4	0.50	NDRG1	4.44
ASNS	0.36	JHDM1D	0.43	CXCR4	0.47	BMI1	0.50	ODAM	4.87

### Functional analysis of TLR9 signaling network in regulation of migration and invasion

**Figure 4:**
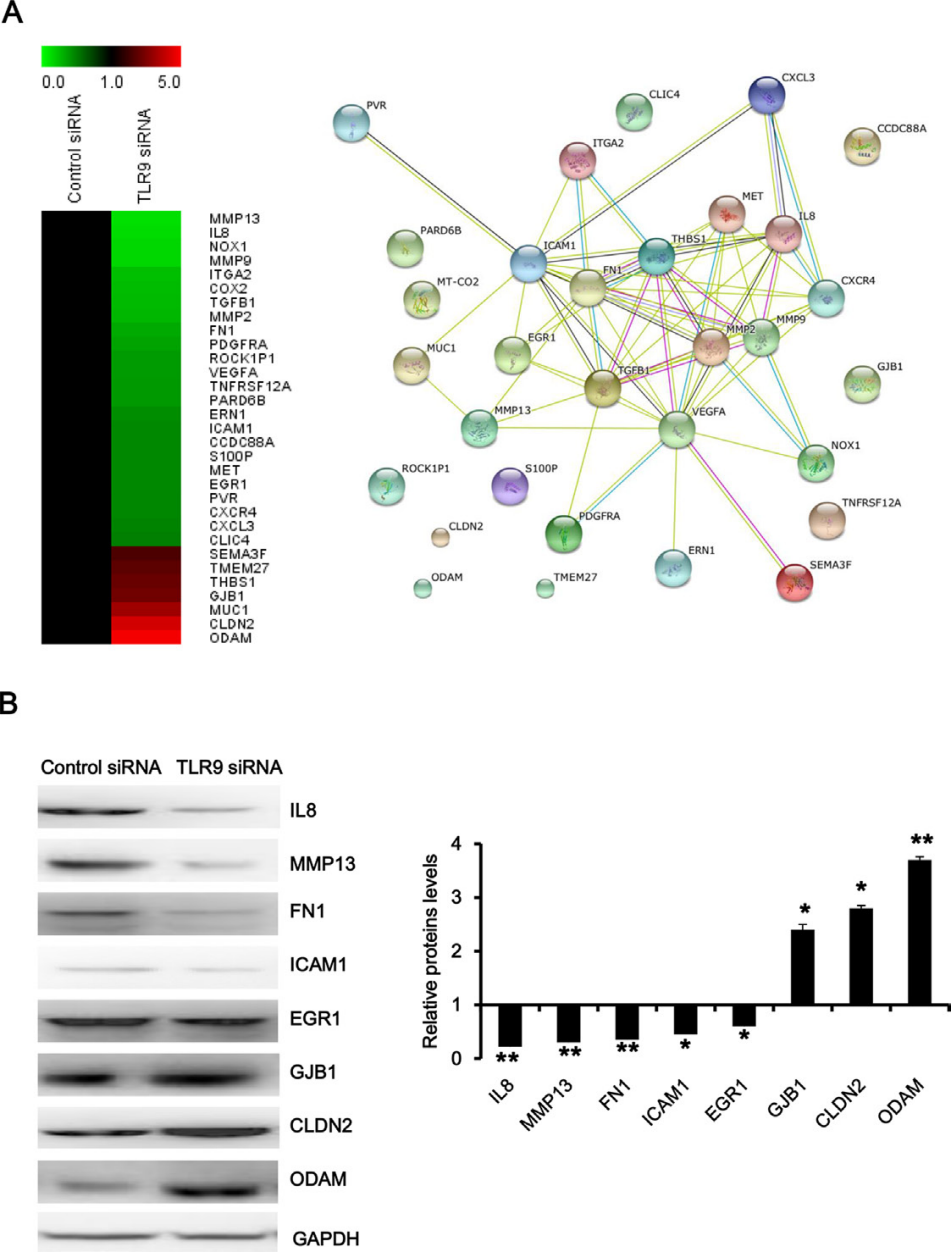
Functional analysis of TLR9 signaling network in regulation of migration and invasion **A.** The heatmap and interaction network of the indicated genes were anaylsed with MeV software and at http://string.embl.de/, respectively. **B.** Proteins were detected by Western blot with the indicated antibodies. The representative figures and quantified data from three individual experiments were shown. * and ** represent *P* < 0.05 and *P* < 0.01, respectively.

To further explore TLR9 signaling network in regulation of migration and invasion of prostate cancer, we focused on analyzing the interactions of genes which might be involved in migration and invasion. As shown in Figure 4A, matrix metallopeptidase 2(MMP2), matrix metallopeptidase 9 (MMP9), chemokine receptor 4 (CXCR4) and interleukin 8 (IL8), chemokine ligand 3 (CXCL3), met proto-oncogene, receptor tyrosine kinase (MET), thrombospondin 1 (THBS1), fibronectin 1 (FN1), integrin alpha 2 (ITAG2), intercellular adhesion molecule 1 (ICAM1), early growth response 1 (EGR1), transforming growth factor beta 1 (TGFB1) and vascular endothelial growth factor A (VEGFA), formed the core interaction network based on their known biological relationships. A few genes, such as odontogenic ameloblast-associated protein (ODAM), claudin 2 (CLDN2), gap junction protein beta 1 (GJB1) and Rho-associated coiled-coil containing protein kinase 1 pseudogene 1 (ROCK1P1), so far have not been found to interact with the other genes. In addition, we validated our microarray results by selection of representative genes, and the results of Western blot experiments showed that silence of TLR9 decreased the expressions of IL8, matrix metallopeptidase 13 (MMP13), FN1, ICAM1 and EGR1 and increased the expression of GJB1, CLDN2 and ODAM (Figure 4B). These results indicated TLR9 regulates the invasion and metastasis of prostate cancer by altering the expression of the aforementioned genes.

## DISCUSSION

Although improvements in molecular diagnostics and targeted therapies were achieved in recent several decades, metastasis of prostate cancer is the main reason for death. New targets are eagerly needed for this disease. Recently, data concerning the overexpresion of TLR9 in a wide variety of cancers including prostate cancer are starting to accumulate [16, 17]. Several clinical studies previously suggest that TLR9 may involving the pathogenesis of various types of cancer, where high expression of TLR9 in tumors is associated with decreased survival in patients with glioblastoma multiforme and esophageal cancer [18, 19]. According to our results, high level of TLR9 expression was significantly correlated with a higher probability of lymph node metastasis, which has been documented to be associated with poor prognosis [20], suggesting TLR9 involving in the metastasis of prostate cancer. Furthermore, we demonstrated that high TLR9 expression in prostate cancer is significantly associated with a decreased b-PFS, indicating the prognostic implications of TLR9 in patients with prostate cancer.

Despite the mechanism of TLR9 promoting prostate cancer has not been totally understood, substantial evidence suggest that stimulation of TLR9 by synthetic DNA ligands or bacterial DNA results in increased capability of cancer cell invasion [21]. Previous studies have also demonstrated that TLR9 expression may regulate cancer cell invasion, even in the absence of ligands in breast cancer cells [22]. Considering that TLR9 was significant in predicting poor prognosis of prostate cancer, we speculated the expression of TLR9 was correlated with the capability of invasion and metastasis of prostate cancer cells. Since Toll-like receptors have been shown to play important roles in host cell responses toward bacteria, bacterial products, fungi, and viruses, these microorganisms, which cause the prostatitis and is recognized by TLRs, is believed to promote chronic inflammation by inflicting cellular damage, cellular hyperproliferation, and increased production of cytokines, which has been associated with prostate carcinogenesis [23]. DNA of microorganisms can be recognized by TLR9 in the endosomal-lysosomal compartment of cells, stimulating inflammation and increasing expression of proinflammatory cytokines and other inflammatory mediators. In our previous research, we have reported that the expression level of proinflammatory cytokines, such as IL-8 and TGF-b1, correlates directly with the metastatic potential of the tumor [24, 25]. In this study, we constructed TLR9-silenced PC-3 cells as a model to investigate the function of TLR9, and the results showed that silence of TLR9 inhibited migration and invasion of prostate cancer.

Although TLR9 regulates tumor invasion in variety of cancer cells, including breast cancer, prostate cancer and colorectal cancer, the signaling pathways of TLR9 in invasion and migration of cancer remains incompletely understood [26]. Several published studies showed that TLR9 signaling could induce the activation of JNK(mitogen-activated protein kinase 8) and p38 MAPK (mitogen-activated protein kinases) pathway to enhance tumor growth [27]. It has been reported that TLR9 signaling could directly promote cancer cell invasion via, at least in part, AP-1 (jun proto-oncogene) activated MMP-2 secretion in HB cells [28]. TLR9 agonist could enhance the metastatic potential of human lung cancer 95D cells via CXCR4/SDF-1(chemokine receptor 4/chemokine ligand 12) pathway [29]. In prostate cancer cells, Ilvesaro et al reported that stimulation of TLR9 with its agonistic CpG-containing ligands increased invasion in human prostate cancer cells by upregulation of MMP13 [15]. Our microarray data implied that TLR9 expression was correlated with regulation of programmed cell death, regulation of locomotion and response to calcium ion, which were believed to have a relationship with cell apoptosis and cell movement. Among 31 identified genes which are related with migration and invasion, some of them, such as MMP13, IL8, and MMP9, have been reported to be downstream molecules of TLR9 signaling pathway [16, 25]. The other genes, like ODAM, CLDN2 and GJB1, have not been reported to have a relationship with TLR9 and others, suggesting they may be new targets for anticancer therapeutic intervention.

In summary, our results demonstrated that overexpression of TLR9 was associated with a higher probability of lymph node metastasis and poor prognosis of prostate cancer. Further silence of TLR9 inhibited migration and invasion of prostate cancer cells. Global gene profiling provides the data about altered gene expressions of TLR9 signaling network in prostate cancer. Functional analysis of TLR9 signaling network in regulation of migration and invasion indentified several genes, which have not been found to interact with the core network consisted of genes involved in migration and invasion. Our data presented here provide important clues for elucidation of the mechanism of TLR9 in invasion and migration of prostate cancer and for development of new anticancer strategies.

## MATERIALS AND METHODS

### Patients and specimens

A total of 78 formalin-fixed, paraffin-embedded specimens is initially collected from prostate cancer patients who underwent radical prostatectomy without neoadjuvant hormonal therapy or transurethral resection of prostate at the Department of Urology, Third Affiliated Hospital of Sun Yat-Sen University, Guangzhou, China from July 2008 to March 2013. Patients with histologically confirmed prostate cancer with diagnostic prostate biopsies and no metastatic disease diagnosed by pelvic computed tomography and bone scan were eligible. The patients were received the radical prostatectomy with standard pelvic lymph node dissection as previously described [30]. Information about Gleason scores (Gleason scoring was only carried out for the prostatectomy samples), clinical stage and prostate-specific antigen (PSA) before surgery were obtained from patient records. The clinical follow-up data include biochemical recurrence (defined as PSA ≥ 0.2 ng/ml on 2 successive measurements in 3 months after radical prostatectomy), clinical recurrence (defined as identification of metastases or histologically confirmed local recurrence) and tumor specific death. Progression-free survival rates were calculated from the date of radical prostatectomy to either biochemical recurrence leading to second line treatments, clinical recurrence or the last day of follow-up. The study was conducted with the ethical approval of the hospital human ethics committee.

### Cell culture and reagents

The human prostate cancer cell line PC-3 was cultured in DMEM with 10% fetal bovine serum, penicillin (100 U/ml) and streptomycin (100 ng/ml) at 37°C under 5% CO_2_ atmosphere in a humidified incubator. Anti-TLR9 (ab37154) and anti-ODAM (ab169694) antibodies were from Abcam. Anti-GJB1 (GTX11368) and anti-CLDN2 (GTX81901) were from GeneTex. Anti-MMP13 (AB20098a) and anti-ICAM1 (AB21861a) antibodies were from Shanghai Sangon Biotech. Anti-IL8 (sc-7922) antibodies were from Santa Cruz Biotechnology. Anti-FN1 (610077) antibody was from BD Bioscience. Anti-EGR1 (4153) was from Cell Signaling Technologies. Anti-GAPDH (KM9002) antibody was from Tianjin Sungene Biotech.

### siRNA transfection

The sense sequences of TLR9 and negative control siRNAs were: 5′-CCGCAUCGUCAAACUGGCG-3′, 5′-CCUACGCCACCAAUUUCGU-3′, respectively, and they were synthesized by Shanghai GenePharma. Each siRNA solution was mixed gently with the respective volume of the X-tremeGENE siRNA Transfection Reagent and allowed to form transfection mixture for 20 mins. Cells were cultured in 6-well plate with DMEM until 50% of confluence and added with the transfection mixture for 48 h before the next experiment [31].

### Western blot analysis

PC-3 cells transfected with control or TLR9 siRNA for 48 h were harvested and lysed in RIPA buffer (1% NP-40, 0.5% sodium deoxycholate, 0.1% SDS, 10 ng/ml PMSF, 0.03% aprotinin, 1 μM sodium orthovanadate) at 4°C for 30 mins. After centrifuged for 10 mins at 14,000 g, supernatants were collected. Protein concentration was quantified using with Bradford assay. Proteins were separated on 12% SDS-PAGE gels and transferred to polyvinylidene difluoride membranes. Membranes were blocked with 5% BSA and incubated with the indicated primary antibodies. Corresponding horseradish peroxidase-conjugated secondary antibodies were used against each primary antibody. Proteins were detected using the chemiluminescent detection reagents and films [32].

### Wound healing assay

PC-3 cells transfected with control or TLR9 siRNA for 48 h was trypsinized and seeded in six-well dishes (5 × 10^5^/well) until the cells reached 80% confluence. The cell monolayer was scratched using a sterile 10 μl pipette tip and washed with PBS three times. Cells were allowed to migrate for 24 h and 48 h in serum-free medium, and the scratches were observed and photographed. The gap lengths were calculated from the photomicrographs.

### Cell invasion assay

Cell invasion assays were performed with a modified Boyden chamber (Corning) containing matrigel-coated polycarbonate membrane filter (6.5 mm diameter, 8 μm pore size) [33]. PC-3 cells transfected with control or TLR9 siRNA for 48 h were plated in the upper chamber and the lower chamber contained medium with 10% FBS, and incubated for 24 h at 37°C in 5% CO_2_. Non-migrated cells were scraped from the upper surface of the membrane, and migrated cells remaining on the bottom surface were photographed and counted.

### Global gene expression analysis

Global gene expression analysis was performed as previously reported [34]. Total RNA was extracted using Trizol reagent from PC-3 cells transfected with control or TLR9 siRNA for 48 h and then was amplified, labeled and purified by using GeneChip 30IVT Express Kit according to the manufacturer's instructions to obtain biotin labeled cRNA. 10 mg of biotin-labelled cRNA (antisense RNA) was hybridized with Affymetrix HTA2.0 array. After hybridization and washing, the fluorescence intensity of the array chips was scanned using a Affymetrix GeneChips Scanner 3000. Raw data were normalized using the MAS 5.0 algorithm, Gene Spring Software 11.0.

### Biological functions and network analysis

The biological network data were generated through the use of with MeV software at http://string.embl.de/, a web-delivered application that evaluates biological networks. A data set of proteins were then used as the starting point for generating biological networks based upon the identities of the focus proteins and interactions with genes-proteins that were reported in the literature. Biological functions or canonical pathways were then calculated and assigned to each network by using findings that had been extracted from the scientific literature. The biological function assigned to each network was ranked according to the significance of that biological function to the network.

### Immunohistochemistry assay

Immunohistochemistry assay was performed with a microwave-enhanced avidin-biotin staining method as previously described [35, 36]. Formalin-fixed, paraffin-embedded tumor tissue slides were deparaffinized using xylene and graded ethyl alcohol and then rinsed in water. Antigen retrieval was performed by boiling the slides in 0.01 M citrate buffer in a microwave oven for 10 mins and cooling at room temperature. The slides were then incubated with 0.05% Triton-X100 in PBS for 5 mins, followed by sequential treatment in a humidified chamber after quenching endogenous peroxides with 3% H_2_O_2_ in MeOH: blocking serum with avidin for 20 mins, anti-TLR9 antibody overnight at 4°C, secondary antibody for 20 mins, hydrogen peroxidase for 15 mins, and peroxidase substrate solution for 20 mins at room temperature. The stained slides were then counterstained with hematoxylin and coverslipped. All sections were evaluated by two independent investigators in a blind manner to reach a consensus. Representative areas were determined to evaluate the expression of TLR9 which was classified as negative (0), weakly positive (+1), positive (+2) or strongly positive (+3). Samples with +2 and +3 staining of TLR-9 were classified as ‘high expression group’, and those with 0 and +1 were assigned as ‘low expression group’.

### Statistical analysis

A student's *t*-test was used to compare individual data points among each group. Correlation of TLR9 expression and clinicopathological variables was analyzed using the χ^2^ test or, in the case of low expected frequencies, by the Fisher's exact test. Progression-free survival rates were calculated using the Kaplan-Meier method, and the statistical significance between groups was analyzed using the log-rank test. Multivariate survival analysis was carried out using the Cox proportional hazards model. Hazard ratios (HRs) were assessed using Cox univariate analysis. *P* < 0.05 was considered statistically significant.
